# Dual Impact of Swimming on Intervertebral Disc Health: Biomechanical, Clinical, and Translational Perspectives

**DOI:** 10.7759/cureus.90474

**Published:** 2025-08-19

**Authors:** Yiqing Ye, Yidong Ye, Jie Zhang

**Affiliations:** 1 Orthopaedics/Physical Medicine and Rehabilitation, Medical University of South Carolina, Charleston, USA; 2 Pharmacology and Immunology, Medical University of South Carolina, Charleston, USA

**Keywords:** artificial intelligence in rehabilitation, intervertebral disc degeneration, low back and neck pain, spinal biomechanics, stroke-specific spinal load, swimming therapy, wearable sensors

## Abstract

Intervertebral disc degeneration (IVDD) is a major contributor to chronic low back and neck pain. Current treatment strategies include conservative pain management and surgical intervention based on clinical presentation. Physical exercise plays an increasingly important role in conservative care, offering both preventive and therapeutic benefits. Among available exercise options, swimming is often considered beneficial due to its low impact nature and supportive biomechanical properties. Its buoyancy reduces spinal load, while water resistance and rhythmic movements help improve core strength, flexibility, and controlled spinal loading. These factors contribute to improved spinal health and support recovery after injury or surgery. However, swimming, especially at a competitive level, also involves repetitive spinal motions such as hyperextension and rotation, potentially increasing the risk of spinal strain or degeneration. This review examines the complex relationship between swimming and IVDD, highlighting both potential benefits and risks. It also discusses stroke-specific considerations, rehabilitation relevance, and personalized recommendations. A translational approach incorporating wearable sensors and intelligent feedback systems is proposed to guide future individualized interventions.

## Introduction and background

**Figure 1:**
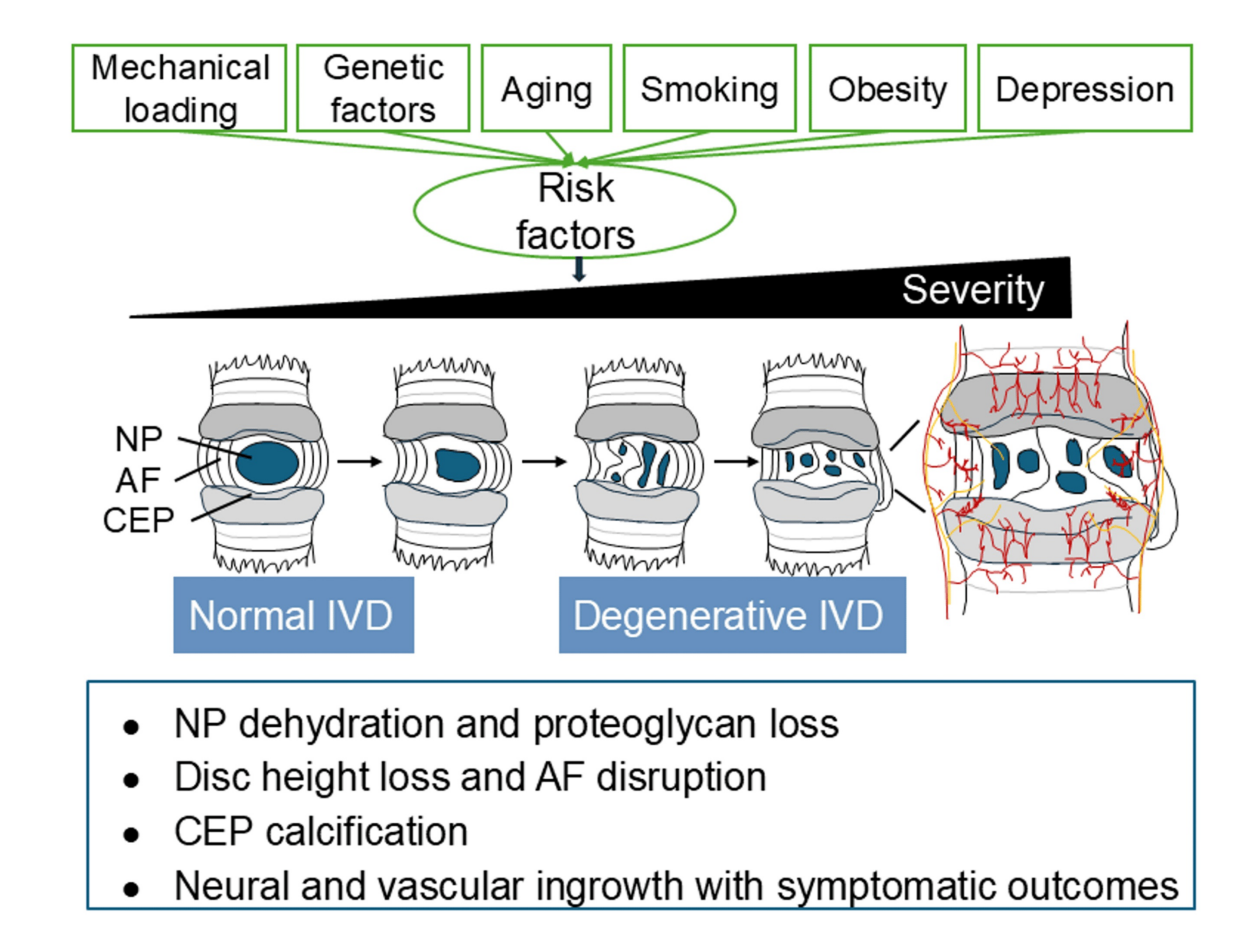
Stages and risk factors of intervertebral disc degeneration The illustration depicts the transition from a healthy intervertebral disc (IVD) to progressive degeneration, influenced by various risk factors, including mechanical loading, genetic factors, aging, smoking, obesity, and depression. Degenerative changes in the IVD are marked by proteoglycan degradation within the nucleus pulposus, resulting in reduced water retention and diminished compressive resistance. These alterations are accompanied by disc height loss, which increases mechanical stress on the annulus fibrosus, promoting annular fissures, herniation, and inflammation. Calcification of the cartilaginous endplates impairs nutrient diffusion and accelerates degeneration. Collectively, these structural deteriorations can compromise disc function, promote blood vessel and nerve ingrowth, and lead to symptoms such as radicular pain, paresthesia, and reduced spinal mobility. Modified from: Orthopedic Clinics of North America, Vol 42, Chan WC, Sze KL, Samartzis D, Leung VY, Chan D, Structure and Biology of the Intervertebral Disk in Health and Disease, Pages 447-464, Copyright 2011 [3]; Permission for use has been taken from Elsevier.

Intervertebral discs (IVDs), located between adjacent vertebrae, play an important role in maintaining the spinal flexibility and ensuring balanced biomechanical loading transmission. Each disc is composed of three regions: the central nucleus pulposus (NP), the peripheral annulus fibrosus (AF), and the cartilaginous endplate (CEP) at the disc-vertebra interface. The NP acts as a cushion to absorb compressive loads, the AF is responsible for tensile strength and structural integrity, and the CEP serves as a semi-permeable barrier facilitating nutrient diffusion and biomechanical conduction (Figure 1) [1]. The integrity of these regions is essential for the homeostasis of the disc, but degenerative changes are common with age, affecting up to 80% of individuals over 50 years of age, with early onset observed in individuals aged 20-40 years [2].

IVD degeneration (IVDD), particularly in the lumbar spine, is a leading cause of low back pain (LBP) and a major public health problem [4]. LBP ranks as the second most common reason for medical consultations and is projected to be the major cause of disability worldwide by 2025 [5]. The economic burden of LBP is substantial, with annual costs over $50 billion and potentially rising to $100 billion under extreme conditions [6]. While lumbar IVDD has been the subject of more research attention due to its well-established association with disability and healthcare burden, cervical disc degeneration is also frequently observed and has important clinical relevance. Chronic neck pain, a common consequence of cervical IVDD, detrimentally impacts quality of life and functional capacity. Occupational factors, including repetitive neck motion, sustained static posture, and mechanical loading, contribute substantially to cervical disc degeneration through biomechanical stress on the spine [7].

IVDD is characterized by the degradation of proteoglycans within the NP, which impairs its water retention capability and compressive resistance. Another feature is the loss of disc height, which leads to mechanical stress on the AF and thereby promotes annular fissures, herniation, and inflammation. Additionally, calcification of the CEP can impair nutrient diffusion and accelerate disc degeneration. These structural deteriorations of the disc can compromise its function and compress neighboring nerve roots, resulting in symptoms such as radicular pain, paresthesia, and reduced spinal mobility [3]. While these pathophysiological changes define the progression of IVDD, its onset and advancement are driven by multiple risk factors. These include non-physiological mechanical loading, genetic factors, aging, and modifiable lifestyle factors such as smoking, obesity, and depression (Figure 1) [8]. 

Despite the significant impact of IVDD on mobility, quality of life, and socioeconomic burden, treatments capable of fully restoring normal disc structure and function remain limited. Management of disease progression typically begins with conservative pain relief strategies, and when the symptoms become severe and significantly impair daily functioning, it may transition to more invasive interventions, such as spinal fusion and/or artificial disc replacement [9]. While surgical and pharmacological treatments remain crucial in advanced stages, non-invasive strategies, particularly physical exercise, are becoming increasingly important in the early and chronic phases, as they are less harmful and aim to preserve function. This review article examines the biomechanical foundation and clinical implications supporting swimming as an intervention for maintaining spinal health and mitigating IVDD. It also discusses the potential for high-intensity swimming to increase IVDD risk, highlighting the need for individualized training to maximize therapeutic benefits while minimizing potential risks. Finally, it proposes a wearable sensor-based concept integrated with artificial intelligence feedback to guide individualized, swimming-based prevention and recovery strategies for IVDD.

## Review

Swimming as a non-invasive modality for IVDD prevention and management

Physical exercise has emerged as a key focus of conservative IVDD management, with both preventive and therapeutic benefits. Exercise-based treatments are recommended in clinical guidelines for improving function, and exercise has been demonstrated to stimulate IVD cell proliferation [10], improve paraspinal muscle strength, and reduce pain and disability [11], particularly when performed with high-load, low-volume, and low-frequency regimens [12]. Such outcomes support its position as a primary, non-invasive intervention for the disc-related conditions.

Among the various forms of physical exercise, swimming is a distinctly advantageous option due to its combined biomechanical and physiological characteristics. It reduces the axial loading, improves muscular strength [13], supports healthy body weight [14-16], enhances circulation [13,17,18], and helps modulate inflammation [13,18], making it suitable for individuals with or at risk of IVDD. Although research on swimming’s impact on IVDD is still under investigation, accumulating clinical and biomechanical evidence suggests its potential to serve as both a therapeutic and a preventative intervention. However, the benefits and risks of swimming for IVDD vary depending on the training intensity, stroke type, and technique. High-intensity training or improper techniques, especially when performing strokes other than freestyle, such as butterfly, may introduce biomechanical stress to predisposed spinal regions. Therefore, individualized training protocols are needed when using swimming for therapeutic purposes. These considerations will be detailed in the following sections.

Mechanobiological advantages of swimming for IVD health

**Figure 2:**
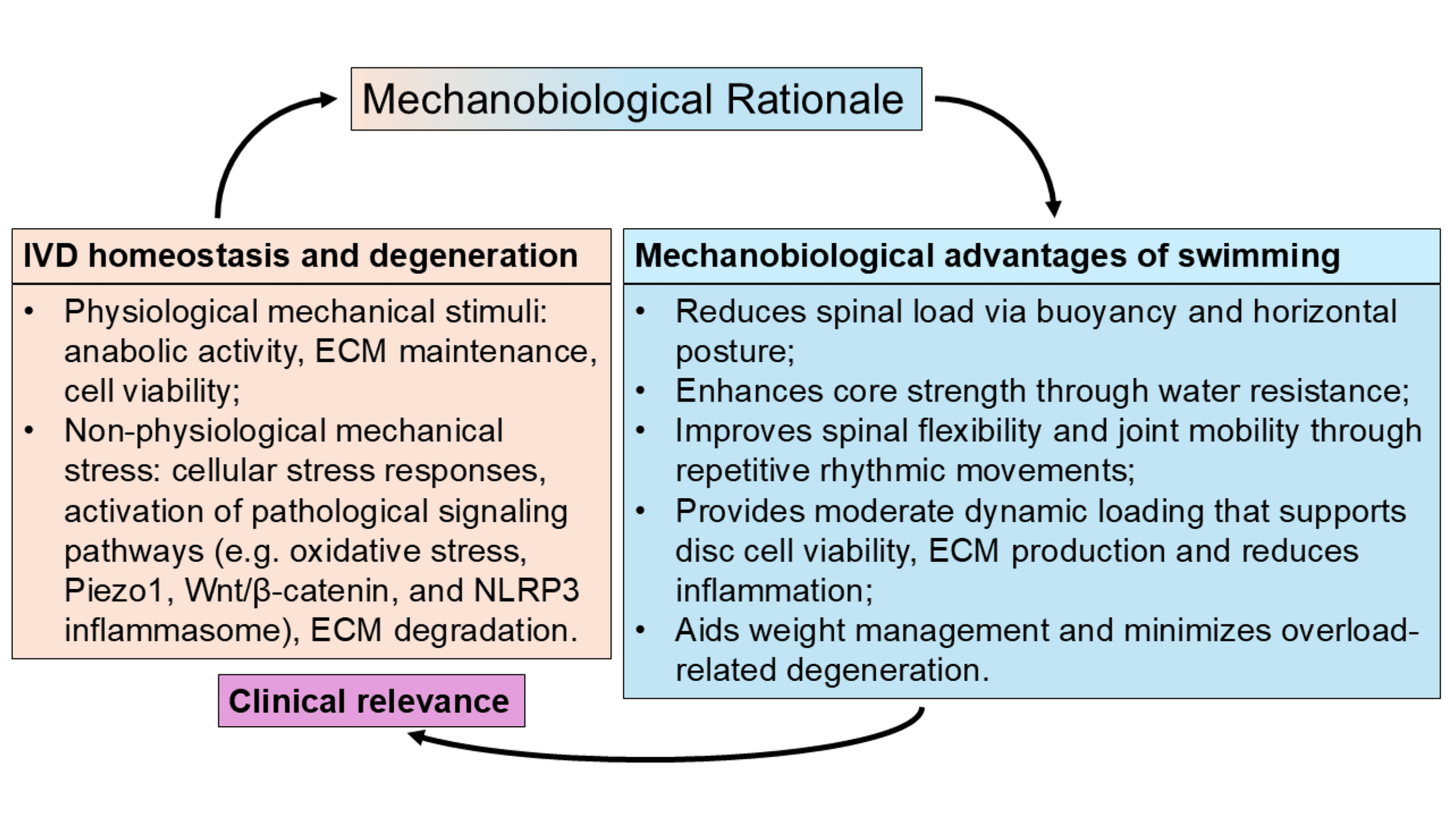
Mechanobiological rationale of swimming for intervertebral disc (IVD) health. Image Credit: Authors Physiological mechanical stimuli promote disc cell anabolic activity, extracellular matrix (ECM) maintenance, and disc cell viability [19], whereas excessive or abnormal loading induces degeneration through cellular stress responses, activation of pathological signaling pathways, and ECM breakdown [8,20,21]. Based on this mechanobiological framework, swimming offers therapeutic benefits by providing controlled, moderate loading in a buoyant environment that reduces spinal compression, enhances core strength through water resistance, improves flexibility and joint mobility through rhythmic movements [13], supports disc cell viability, ECM production while suppressing inflammation [8,19,21], and aids in weight management to minimize overload-related degeneration [14-16]. ECM: extracellular matrix

IVD homeostasis and degeneration are highly influenced by mechanical loading (Figure 2). Physiological levels of mechanical stimulation (e.g., low-to-moderate static compression, hydrostatic pressure, or osmotic stress) lead to stimulation of anabolic processes in IVD cells, especially NP and inner AF, which support extracellular matrix (ECM) maintenance and cell survival [19]. Conversely, non-physiological mechanical stress (e.g., excessive magnitude, abnormal directionality, or prolonged static loading) perturbs disc homeostasis by initiating a chain of detrimental cellular responses, such as endoplasmic reticulum stress, mitochondrial damage, apoptosis, ferroptosis, senescence, autophagy, inflammation, and ECM degradation. These pathological responses are regulated by mechanosensitive signaling pathways such as Piezo1, Wnt/β-catenin, and NLRP3 inflammasome [8]. Reactive oxygen species overproduction-induced oxidative stress are the upstream amplifiers of these responses, linking mechanical overload with redox imbalance, further promoting degenerative cascades of IVD cells [20]. The existence of such a mechanobiological threshold is further supported by Wang et al., who reported that moderate cyclic tensile stretch (5%) of AF cells stimulates YAP and represses the NF-κB pathway, which leads to a decrease in inflammatory levels and an increase in ECM synthesis and cell proliferation [21], whereas overloading (12%) leads to YAP inactivation, increased NF-κB signaling, inflammation and catabolic responses. In vivo evidence also supports that moderate mechanical loading brings disc structure back and increases ECM in degenerated discs [21]. Together, these findings highlight the therapeutic potential of mechanical modulation in IVDD and help to emphasize the importance of retaining loading in the physiological window to maintain disc integrity.

Conversely, non-physiological mechanical stress (e.g., excessive magnitude, abnormal directionality, or prolonged static loading) perturbs disc homeostasis by initiating a chain of detrimental cellular responses, such as endoplasmic reticulum stress, mitochondrial damage, apoptosis, ferroptosis, senescence, autophagy, inflammation, and ECM degradation. These pathological responses are regulated by mechanosensitive signaling pathways such as Piezo1, Wnt/β-catenin, and NLRP3 inflammasome [8]. Reactive oxygen species overproduction-induced oxidative stress are the upstream amplifiers of these responses, linking mechanical overload with redox imbalance, further promoting degenerative cascades of IVD cells [20]. The existence of such a mechanobiological threshold is further supported by Wang et al., who reported that moderate cyclic tensile stretch (5%) of AF cells stimulates YAP and represses the NF-κB pathway, which leads to a decrease in inflammatory levels and an increase in ECM synthesis and cell proliferation [21]. Whereas overloading (12%) leads to YAP inactivation, increased NF-κB signaling, inflammation, and catabolic responses. In vivo evidence also supports that moderate mechanical loading brings disc structure back and increases ECM in degenerated discs [21]. Together, these findings highlight the therapeutic potential of mechanical modulation in IVDD and help to emphasize the importance of retaining loading in the physiological window to maintain disc integrity.

Taking this mechanobiological paradigm into account, swimming emerges as a promising form of exercise that provides suitable mechanical stimuli to support spinal health (Figure 2). As a type of aquatic exercise, swimming involves dynamic, whole-body movement in a buoyant environment [13]. At varying depths of immersion, water can reduce effective body weight to near-negligible values, thus minimizing compressive forces on spinal structures and enabling spinal unloading, while still allowing active muscle engagement. The horizontal body position in the water during swimming results in an equal distribution of mechanical load across the spine, in contrast to upright land-based activities that can put excessive stress on the intervertebral discs. Additionally, water viscosity offers natural resistance, which stimulates spinal stabilizers (e.g., paraspinal and abdominal muscles). This neuromuscular activation increases the core strength and postural control; both are necessary to support the stability of the spine of individuals with IVDD. The repetitive rhythmic movements of swimming also help foster spinal flexibility and joint mobility, potentially reducing stiffness and loss of movement often found to accompany disc degeneration. By integrating spinal unloading with regulated muscle-activating movement, swimming reproduces the moderate and dynamic loading conditions that have been reported to maintain disc cell viability, promote ECM production, and inhibit inflammatory responses [8,19,21].

Furthermore, swimming and aquatic exercise serve as effective strategies for weight management [14], a critical factor given that obesity and excess body weight are known comorbidities of lumbar IVDD [15,16]. Reducing body weight mitigates chronic mechanical overload to spinal motion segments and may help slow disease progression. As swimming avoids the excessive mechanical loading that induces degenerative cascades, it may help maintain disc health in asymptomatic individuals and reduce matrix catabolism and inflammation in early degenerating states. While these characteristics overlap with the general advantages outlined in Section 1, here they are considered through the lens of mechanobiology, where swimming represents a non-invasive strategy for the prevention and conservative management of IVDD.

Physical and clinical benefits of swimming for IVDD

**Figure 3:**
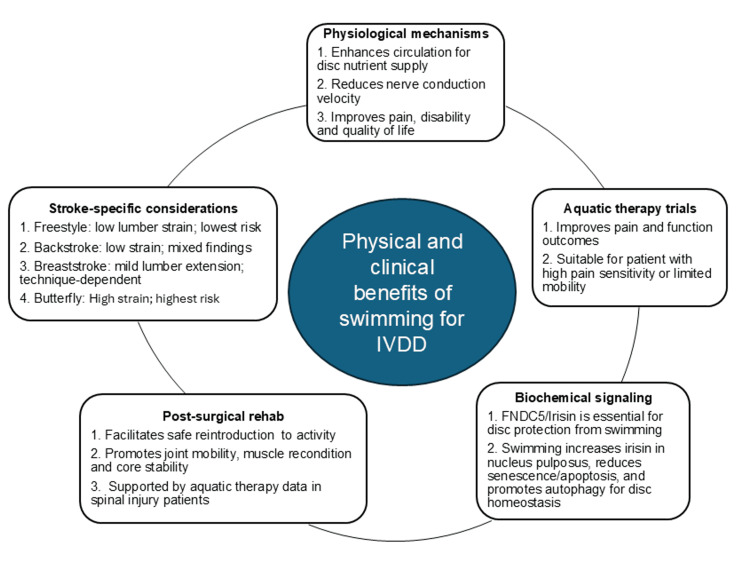
Physical and clinical benefits of swimming for intervertebral disc degeneration (IVDD) Swimming and aquatic therapy improve outcomes in IVDD through complementary physiological, biochemical, and rehabilitative mechanisms. Physiological benefits include enhanced systemic circulation for disc nutrient delivery, reduced nerve conduction velocity, and improvements in pain, disability, and quality of life [13,17,18]. Clinical trials have demonstrated superior pain relief and functional gains in patients with high pain sensitivity or reduced mobility [22-24]. At the biochemical level, swimming increases the expression of FNDC5/irisin in the NP. This exercise-induced myokine reduces cellular senescence and apoptosis while promoting autophagy, a key process for disc cell protection and tissue homeostasis [25]. Swimming may also offer benefits in post-surgical spinal rehabilitation. It facilitates a safe reintroduction to physical activity, promotes joint mobility, muscle reconditioning, and core stability, and is supported by evidence from aquatic therapy studies in patients with spinal injuries [26]. Stroke-specific considerations are essential. Freestyle and backstroke are symmetrical, long-axis strokes generally associated with lower lumbar strain and reduced risk of IVDD, though findings for backstroke are mixed. Breaststroke and butterfly are horizontal-axis strokes. Breaststroke moderately increases lumbar extension and requires proper technique to avoid strain, while butterfly carries the highest risk due to its high intensity and repetitive lumbar hyperextension [27-29]. Image Credit: Authors

Consistent with the biomechanical rationale provided, clinical studies also indicate that swimming and aquatic therapy are valuable modalities for spinal health, IVDD management, and chronic LBP rehabilitation (Figure 3). 

Physiological Mechanisms Supporting Disc Health

Physiologically, swimming improves systemic circulation [13,18], resulting in nutrient flow to the predominantly avascular intervertebral discs necessary to maintain disc cell viability and matrix integrity [17]. Furthermore, the thermal and hydrostatic characteristics of the aquatic environment have been shown to reduce nerve conduction velocity and pain perception, accounting for the analgesic effects of hydrotherapy [18]. From a clinical perspective, these physiological benefits are reflected in marked improvements in pain, disability, and quality of life.

Evidence from Aquatic Therapy Trials

Numerous clinical trials have shown that aquatic therapy is an effective treatment for chronic LBP, especially in patients with impaired mobility or high sensitivity to pain. In a randomized controlled trial (RCT), Dundar et al. demonstrated that participants in the aquatic exercise group experienced significantly greater reductions in pain intensity and disability, as well as improved physical output compared with those in a land-based program [30]. Similarly, Waller et al., in a systematic review, found that aquatic therapy was more effective in pain and functional improvement, especially in individuals with limited tolerance for weight-bearing activities [22]. These findings were supported by a meta-analysis of Shi et al., which confirmed the benefits of aquatic exercise for LBP patients, although improvements in subjective psychological symptoms were less consistent [23]. Ma et al. also substantiated the clinical efficacy of hydrotherapy, reporting greater improvements in pain and quality of life compared with land-based therapy [24]. Although these studies provide evidence supporting the benefits of swimming for rehabilitation, the quality of evidence presented overall appears to be low. Current conclusions are weakened by methodological limitations, including small sample size, variability in the intervention protocols, and short follow-up periods. Thus, while the benefits described here align with those summarized earlier, these studies provide direct clinical evidence and highlight the need for standardized, high-quality RCTs with long-term follow-up.

Swimming-Induced Biochemical Signaling and Cellular Protection

While physical exercise has been shown to delay the progression of IVDD, the underlying biochemical signaling is not fully understood. In a recent study using rodent models, the impact of swimming on IVDD was studied [25]. Moderate daily swimming significantly increased the irisin (an exercise-induced myokine encoded by the *FNDC5* gene) levels in the blood circulation and in the NP tissue. Such an increase in irisin levels is related to a reduction in cellular senescence and apoptotic markers of NP cells, as well as to an increase in autophagy, a critical cellular process to maintain homeostasis and tissue health. Notably, mice deficient in *FNDC5* did not show the protective effects of swimming, indicating the critical role of irisin in exercise-induced spinal protection. These data suggest that swimming may attenuate or delay IVDD through irisin-mediated mechanisms, offering a mechanistic complement to the clinical benefits already outlined.

Post-Surgical Rehabilitation Applications

Following spinal procedures, such as microdiscectomy or spinal fusion, swimming may be a viable rehabilitative option. Once the acute period of recovery is finished and the patient is medically cleared, it provides a way to gradually reintroduce physical activity. The buoyancy of water reduces axial loading on the spine, enabling joint mobilization and muscular reconditioning with minimal mechanical stress on healing tissues. Although direct clinical studies on swimming in postoperative spinal patients are limited, evidence from aquatic therapy in populations with spinal cord injury supports its utility in functional recovery [26]. Here, the advantage is less about general unloading (as noted earlier) and more about enabling safe, progressive rehabilitation during tissue healing.

Swimming as a Clinical Modality: Stroke-Specific Considerations

Swimming possesses several rehabilitative characteristics similar to those observed in structured aquatic therapy. As outlined earlier, its low-compression nature and spinal unloading allow individuals to execute movements that may be painful or biomechanically impossible on dry land. A previous review, which included 121 articles on physical activity recommendations for individuals with chronic LBP after completing rehabilitation, highlighted the benefits of low-compressive aerobic exercises such as swimming, walking, and cycling [27]. These exercises help maintain fitness and flexibility while reducing mechanical stress on the spine. Freestyle, backstroke, and breaststroke are regarded as favorable strokes due to their symmetrical movement and relative safety for the spine. In contrast, butterfly is deemed unsuitable because of its high intensity and the potential for lumbar hyperextension, which can exacerbate symptoms or lead to structural injuries, such as spondylolysis. This underlines the importance of good stroke technique and proper intensity adjustment, especially for individuals with spinal vulnerabilities.

Building on these results, a scoping review analyzed 25 studies (16 observational, five interventional, and three biomechanical) to investigate the role of swimming in LBP management [28]. Observational studies consistently describe leisure swimming as low-risk and potentially beneficial for individuals with LBP or disc degeneration. Moderate-level recreational swimming appears to be independently associated with improved spinal mobility and fitness, whereas high-intensity swimming at competitive levels over long periods of time may increase the risk of LBP or disc pathology. Interventional studies tentatively support incorporating swimming into organized rehabilitation programs, as they demonstrate more favorable pain and function outcomes. However, considerable variability in study design, intervention duration, and outcome measures prevents definitive conclusions. Biomechanical analyses provide additional insight into the impact of different strokes on spinal mechanics. Long-axis strokes like freestyle and backstroke involve longitudinal body rotation, resulting in less lumbar strain and lordosis compared to standing upright. On the contrary, the horizontal axis movement in breaststroke slightly increases lumbar extension. While generally safe, improper techniques such as maintaining the head above water or excessive undulation can worsen symptoms, particularly in patients with existing spinal conditions.

Aligned with these findings, another scoping review analyzed 44 studies on swimming and LBP and pointed out that freestyle is consistently associated with reduced risk (odds ratios 0.04-0.74), while butterfly is linked to a significantly higher risk (odds ratios 0.97-2.7) [29]. Backstroke and breaststroke show mixed results, emphasizing the need for individualized stroke selection and proper technique.

Collectively, these reviews indicate that despite common clinical recommendations and biomechanical reasoning for swimming as a therapeutic intervention for LBP, the evidence is both limited and of poor quality. Nonetheless, swimming is a safe and potential option, especially in personalized, multidisciplinary rehabilitation protocols. Prospective and robust RCTs are needed to validate stroke-specific guidelines and explore swimming's clinical implications in preventing and managing spinal pathologies.

Risks and contraindications of swimming in IVD health

**Figure 4:**
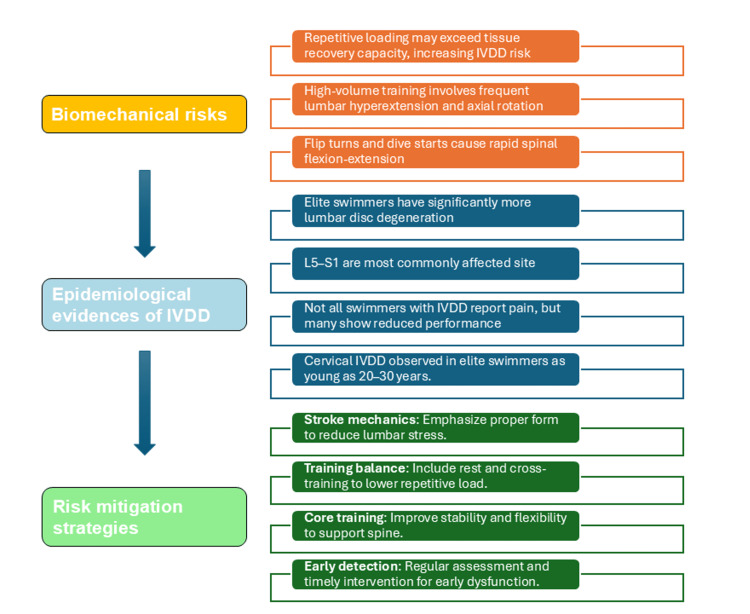
Risks of IVDD in competitive swimmers Image Credit: Authors In competitive swimmers, swimming may increase lumbar disc stress through repetitive spinal motions, including excessive lumbar hyperextension and axial rotation, as well as additional biomechanical loads from flip turns and dive starts involving rapid flexion-extension movements [31]. Epidemiological studies show increased prevalence of lumbar (L5-S1) [31-33] and cervical disc [34] degeneration in elite swimmers, appearing even at younger ages [35]. These changes may be symptomatic or asymptomatic but often impact performance and spinal health. Proper technique, balanced training, core training, and early intervention are essential for risk mitigation [31]. IVDD: intervertebral disc degeneration

While recreational swimming offers therapeutic potential, growing evidence, including biomechanical analyses and epidemiological data, shows a higher prevalence of IVDD in competitive swimmers (Figure 4). It is important to emphasize that these biomechanical risks are primarily observed in elite or high-volume swimmers, and not in the general population. For most recreational swimmers, especially those engaging in moderate-intensity freestyle, swimming remains a safe and beneficial activity for spinal health.

Biomechanical Risks in Competitive Swimming

Although swimming is generally regarded as a spine-friendly activity, evidence suggests that it may not always be beneficial for your spine, especially with high levels of training. A recent systematic review from Hsu et al. highlights this paradox [31]. While swimming reduces the gravitational loading on the spine (an important cause of IVDD) through water buoyancy, it may potentially impose additional stress on discs. Repetitive spinal motions, particularly lumbar hyperextension and axial rotation during strokes like butterfly and breaststroke, may predispose swimmers to IVDD. Furthermore, biomechanical stressors, such as flip turns and dive starts involving rapid spinal flexion and extension, may cumulatively aggregate the loading on the lumbar spine.

Epidemiological Evidence of IVDD in Swimmers

Lumbar IVDD and LBP prevalence is considerably higher among elite swimmers when compared to recreational swimmers and non-athletes [31]. The high intensity levels and high training volume in competitive swimmers seem to be one of the critical contributors to disc degeneration in these individuals. The L5-S1 discs are particularly at risk. In a case-control study, degenerative changes at those discs were observed in 68% of the elite swimmers compared to 29% of the recreational swimmers, identifying L5-S1 as the most frequently affected region [32].

Although some athletes with disc degeneration are asymptomatic, others have experienced moderate to severe back pain, which detrimentally affects athletic performance and quality of life. A cross-sectional analysis found that despite similar prevalence of IVDD and back pain between elite swimmers and non-athletes, the anatomical location differed [33]. For instance, elite swimmers exhibited more degeneration in the upper lumbar spine. Similarly, data from the Rio 2016 Olympic cohort suggested that disc degeneration was frequently observed across several sports, with swimming, athletics, and boxing being most commonly associated with heavy degenerative changes in the cervical spine [34]. Critically, swimmers showed signs of cervical IVDD at the age of 20-30 years, suggesting that repetitive extension and axial loading in competitive swimming may lead to accelerated degeneration of the IVD [35].

Additional support is provided by a large cross-sectional study that compared the prevalence of IVDD between different sports [36]. Athletes involved with competitive swimming and baseball were more likely to have lumbar disc degeneration than non-athletes. This study also demonstrated a linear relationship between lifetime back pain and disc degeneration, suggesting clinical symptomatology as a predictor of the underlying structural alteration.

Importantly, these concerns are not limited to elite athletes. In a population-based MRI cohort study involving 558 adolescents aged 20-22 years, sports participation was self-reported by postal questionnaire when they were at ages of 16, 18, and 19 years [37]. Swimming was one of the sports evaluated for its long-term effects on lumbar disc health. Active adolescents who swam a minimum of twice a week showed significantly higher lumbar degeneration scores than their inactive counterparts (swam at a maximum of once a month). This pattern remained after controlling for gender, age, BMI, smoking, socioeconomic status, and involvement in other sports, indicating that performing repeated spinal extension and axial loading early in life might be followed by long-term consequences.

Optimizing swimming for IVD health: from risk mitigation to translational innovation

Swimming has both positive and negative impacts on the health of IVD. On one hand, swimming provides controlled mechanical stimuli, spinal unloading, and muscular activation under physiological conditions that may contribute to disc cell viability, extracellular matrix homeostasis, and functional stabilization. These outcomes are supported by clinical observations of reduced pain, improved flexibility, and restored physical function among patients with IVDD and LBP following swimming and aquatic therapy. On the other hand, if performed excessively or incorrectly, particularly at competitive levels or through the application of biomechanically unfavorable strokes such as butterfly (with mixed results for backstroke and breaststroke), swimming may become an additional source of spinal loading, leading to an increased propensity for wear and tear in susceptible regions such as the lumbar L5-S1 and the cervical discs.

Risk Mitigation Strategies

Given the dual effects, a multifaceted individualized approach is necessary to preserve the benefits while limiting the risks of swimming: (i) Stroke mechanics optimization: An emphasis on technique should be made to minimize lumbar hyperextension and axial torsion, (ii) Training modifications: Sufficient rest time and alternative cross-training activities should be included to reduce repetitive spinal loading, (iii) Core stability and flexibility: Making core musculature stronger and more flexible may protect the spine, and (iv) Surveillance and early intervention: Monitoring of musculoskeletal health with appropriate intervention at early indication of dysfunction could potentially intercept the development of sub-clinical pathology (Figure 4) [31]. These support the requirement for individualized swim interventions performed according to spinal health and overall biomechanical risk.

Translational Perspective

To systematically optimize the clinical application of swimming in IVDD, future research should focus on the following three domains of specialization.

Biomechanical characterization: Fine-grained, high-resolution kinematic and electromyography (EMG) studies are required to quantify the spinal loading patterns during different strokes, intensities, and durations of swimming. Knowing the stroke-specific mechanical thresholds that differentiate beneficial from harmful loading will be crucial for evidence-based guidelines.

Clinical standardization: Robust and large-scale RCTs need to be performed to evaluate the effectiveness and safety of stroke-related swimming interventions in the prevention and recovery of IVDD. Such studies must include standardized outcome measures, long-term follow-up, and patient stratification based on underlying spinal diagnosis and risk factors.

Translational significance: To integrate biomechanical understanding into personalized treatment, we propose the design of wearable devices (e.g., waterproof collars or belts containing surface EMG electrodes and inertial sensors) that can record the trunk muscle activities and kinematics during swimming. When combined with AI-based algorithms, such technologies could provide real-time feedback, identify maladaptive biomechanics (e.g., excessive lumbar extension), and offer personalized cues for correction. This is a novel approach; only one Initial study on intelligent swimming analysis has demonstrated the potential for stroke classification and biomechanical modeling with wearable devices [38]. Realization of this vision requires a multidisciplinary approach, which will involve biomechanists, engineers, rehabilitation experts, and clinicians. Mechanobiological studies that seek to model the system in a quantitative manner will need to be translated into threshold-based feedback algorithms. At the same time, sensor-guided swimming should be integrated into rehabilitation protocols as part of clinical care and be safely adapted for both prevention and recovery. Clinician participation will be crucial for confirming the feasibility and utility of these strategies in routine practice. The wearable sensor and AI-guided feedback concept presented here is intended as a translational research direction rather than an immediate clinical recommendation. Its efficacy, feasibility, and cost-effectiveness must be validated in future large-scale studies before being implemented in general swimming programs. Such technology may be most appropriate for high-risk subgroups rather than the general swimming population.

## Conclusions

Swimming represents a biomechanically distinct form of physical activity with context-dependent effects on IVD health. While moderate-intensity recreational styles remain broadly beneficial for most individuals, certain high-intensity training contexts and biomedically demanding strokes may increase risk in susceptible populations. Rather than applying a uniform prescription, its use in IVDD prevention or rehabilitation should be guided by individualized assessment of spinal condition, stroke-specific loading patterns, and overall risk factors. Emphasis should be placed on aligning biomechanical demands with the disc’s physiological tolerance to promote long-term spinal integrity. Interdisciplinary efforts integrating clinical insight, biomechanical modeling, and wearable sensor technologies hold promise for advancing swimming-based interventions. Such innovations, once validated through high-quality studies, may enable real-time monitoring and personalized feedback, ensuring safer and more targeted application of aquatic exercise across both clinical and athletic populations.
